# CAMM Techniques for the Prediction of the Mechanical Properties of Tendons and Ligaments Nanostructures

**DOI:** 10.1100/tsw.2005.77

**Published:** 2005-07-22

**Authors:** Simone Vesentini, Franco M. Montevecchi, Alberto Redaelli

**Affiliations:** ^1^ Department of Bioengineering, Politecnico di Milano, Via Golgi 39 20139, Milan, Italy; ^2^ Department of Mechanics, Politecnico di Torino, Corso Duca degli Abruzzi, 24 10129, Turin, Italy

**Keywords:** tendon, ligament, extracellular matrix, collagen molecule, collagen fibril, proteoglycan, decorin, glycosaminoglycan

## Abstract

Theoretical prediction of the mechanical properties of soft tissues usually relies on a top-down approach; that is analysis is gradually refined to observe smaller structures and properties until technical limits are reached. Computer-Assisted Molecular Modeling (CAMM) allows for the reversal of this approach and the performance of bottom-up modeling instead. The wealth of available sequences and structures provides an enormous database for computational efforts to predict structures, simulate docking and folding processes, simulate molecular interactions, and understand them in quantitative energetic terms. Tendons and ligaments can be considered an ideal arena due to their well defined and highly organized architecture which involves not only the main structural constituent, the collagen molecule, but also other important molecular “actors” such as proteoglycans and glycosaminoglycans. In this ideal arena each structure is well organized and recognizable, and using the molecular modeling tool it is possible to evaluate their mutual interactions and to characterize their mechanical function. Knowledge of these relationships can be useful in understanding connective tissue performance as a result of the cooperation and mutual interaction between different biological structures at the nanoscale.

Theoretical prediction of the mechanical properties of soft tissues usually relies on a top-down approach. That is analysis is gradually refined to observe smaller structures and properties until technical limits are reached. Computer-Assisted Molecular Modeling (CAMM) allows the performance of bottom-up modeling instead, that is starting from nanoscale structures to gain microscale information and ultimately to relate the hierarchical structure with the mechanical performance. For this purpose tendons and ligaments can be considered an ideal arena due to their well defined and highly organized architecture which involves not only the main structural constituent, the collagen molecule, but also other important molecular "actors" such as proteoglycans (PGs) and glycosaminoglycans (GAGs). In this ideal arena each structure is well organized and recognizable, and using the molecular modeling tool it is possible to evaluate their mutual interactions and to characterize their mechanical function.

Summing up the most recent outcomes in this area devoted to the understanding of the mechanical behavior of a tissue as a result of its nano-features, it is possible to define that tendons and ligaments are hierarchically organized and that the main structural building block is the collagen molecule which auto-assembles, starting from the bottom, in microfibrils and fibrils. Fibrils themselves come together to form tendon fiber and ultimately the whole tendon. These structures are not continuous; in fact collagen molecules are shorter than the fibril that they form, and fibrils are shorter than the fiber that they form. This observation has a direct consequence if we intend to investigate the mechanical behavior of such a system. In fact, from a mechanical standpoint there is no requirement that the inferior structure should extend from one end of the higher structure to the other. The only requirement is that a linkage between inferior structures should exist. Concerning this hypothesis, the role of GAGs' chains was previously suggested[1] successively experimentally observed[2,3,4] and recently computationally investigated[5,6]

Concerning the role of PGs, up to the middle eighties Professor Scott proposed a mechanical role of these structures and introduced a "shape modulus" composed of collagen fibrils linked by PGs which would behave like stress transfer structures between contiguous fibrils[1]. Decorin is the above mentioned PG which is found linked to fobrillar surface every 68 nm along the fibril, the linkage is due to the shape of this molecule which is extremely and amazingly complementary with a collagen molecule. Recently, Professor Iozzo's research group provided crucial works concerning the tertiary structure of the decorin core protein[7] and the location of the decorin binding region along the collagen molecule[8]. These studies represented the starting point for our molecular studies. The role of GAGs' chains as stress transfer structures along fibrils was investigated from the mechanical standpoint in 2003[5]. Successively, the mechanical response of collagen molecule sequences due to elongation was estimated[9] and at the same time the interaction between collagen molecule and decorin was evaluated[6] in order to characterize the mechanical performance of this complex which seemed a crucial ring in this molecular system. In fact, the properties of the bond between the decorin core protein and collagen are essential in determining the overall feasibility of the GAG chain to behave like a stress transfer structure between collagen fibrils (Figure 1). The bond must be sufficiently strong and stiff in comparison to the GAG chains to be able to transfer mechanical stress from fibril to the GAGs and vice versa.

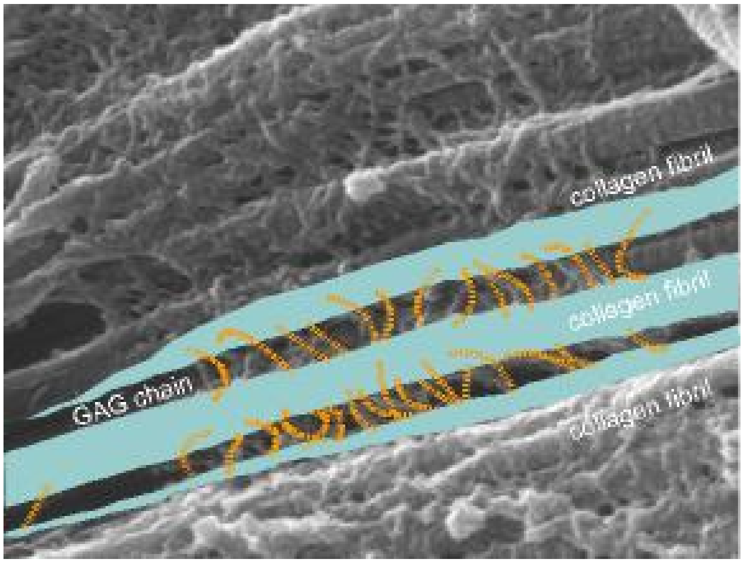

**FIGURE 1.** ***Scanning electron micrograph of rat tail tendon.*** The linkage between adjacent fibrils, mediated by GAGs' chains, is shown. The thick interweaving of collagen fibrils (green) and GAGs' chains (orange), responsible for distributing the mechanical loads across the whole tissue, is also delineated. The horizontal field of view spans 2 μm[10] .

In particular, our study recently published in the Journal of Biomechanics[6] was aimed at the evaluation of the interaction energy and ultimately the binding stiffness between two molecular structures within tendon and ligaments; namely the type I collagen molecule and the decorin core protein, the main proteoglycan (PG) in tendon and ligament ECM. The binding stiffness is a mechanical parameter which is deeply related to the affinity of a molecular complex. In order to characterize the binding stiffness of this complex the molecular mechanics approach was used. This approach required the definition of the molecular structures involved. Concerning the collagen molecule, the knowledge of its primary sequence is the starting prerequisite. Type I collagen molecule consists mainly of two $\alpha_1$(I) chains and one $\alpha_2$(I) chain. The entire primary sequence for human collagen type I can be obtained from the online GenBank database (entry number for the $\alpha_1$(I) and $\alpha_2$(I) chains are NP_000079 and NP_000080, respectively). GenBank is the NIH genetic sequence database, an annotated collection of all publicly available DNA sequences with their correspondent amino-acid sequence. The secondary and tertiary structures for molecular mechanics simulations were generated in the PDB file format starting from the primary sequence and using software developed by Rainey and Goh[11]. Generally speaking, it would be possible to build the wide length of the helical region within the collagen molecule (more than 1000 residues), but in order to reduce computational costs, small oligopeptides or subdomains (30 resides long) can be investigated if sufficiently longer than the dimension of the decorin inner region. Moreover, despite its minor abundance, the homotrimer form was adopted. Following these simplifications and assumptions, and focusing our attention on the collagen sequence which Iozzo's research group indicated to be placed at the decorin binding site[8] the binding stiffness was calculated and confronted with another sequence placed away from this site. Concerning the molecular model of the decorin core protein, its primary structure is known[12], and only recently its secondary and tertiary structure was clarified. In 1996, the crystal structure of the porcine RNAse inhibitor was used to achieve the decorin upper level structures[7] due to the observation that these two molecules have a structural homology[13]. Following this conclusion, Iozzo's research group used the crystal structure of the porcine ribonuclease inhibitor, -PDB identification code 1DFJ-, to build the decorin core protein[7]. The porcine ribonuclease inhibitor had served as a well-accepted model for the structure of decorin for years. Recently, a paper on the crystal structure of dimeric decorin was published[14]-PDB identification code

1XKU-. Each monomer is a single-domain structure with the right handed curved solenoid fold characteristic of leucine rich region proteins giving rise to a “banana” shape. Undoubtedly, the ‘‘horseshoe’’-like structure of RNAse inhibitor differs from the more opened ‘‘banana’’-shaped molecules. Concerning this outcome, Iozzo’s group suggested that decorin is a monomer solution and that the dimer is an artifact of dialysis and lyophilization[15]). Nowadays, this quarrel remains not completely resolved. In general NMR and X-ray methods are used to determine the structure of a given protein; the output generates co-ordinate data that are usually deposited in the Protein Data Bank (or PDB, web site http://www.rcsb.org/pdb/). These experimental methods are obviously more realistic than molecular design techniques and the number of resolved structures is growing rapidly. This was also the case of decorin tertiary structure which was published a few months after our paper was accepted for publication. Interestingly, comparing the molecular model proposed by Iozzo with the dimeric crystal structure by Scott, the 10 leucine rich regions align very well and the inner curvature is nearly identical. This remark makes us confident that if only this concave surface interacts with the collagen molecule (obvious in the monomeric form, unclear in the dimeric form) our assumption, that is that the RNAse inhibitor tertiary structure can be used as a blueprint, seems plausible. Undoubtedly, a more realistic model should take into account all the decorin assembly. In our work, the primary sequence of the RNAse inhibitor was exchanged with the human decorin sequence, and the decorin tertiary structure in a monomer conformation was achieved and subsequently energetically minimized through the molecular mechanics approach in order to obtain the equilibrated configuration of the decorin core protein.

The interaction between the PGs and collagen appears to be specific and mediated largely by the inner domains. Decorin can accommodate a collagen molecule in its cavity and is thought to bind to collagen via the β-sheet regions that line the inner surface of the horseshoe. Next, the interaction energy of both stretches of collagen was hence evaluated stepwise as function of the intermolecular distance. Figure 2 shows five different conformations of the molecular system in the interaction energy curve of this complex.

The interaction energy was defined as the difference between the total energy of the decorin-collagen assembly and the energies of the two separate structures. The curve of (Figure 2) was obtained by optimizing this complex. Each optimization was stopped when the potential energy gradient became lower than 0.001 kcal/Å·mol. For each intermolecular distance, the interaction energy ($E'_{DC}$) was obtained by subtracting the collagen ($E_C$) and the decorin ($E_D$) potential energy from the bulk system potential energy ($E_{TOT}$). Afterwards, the interaction energy $E'_{DC}$ was interpolated using a Lennard-Jones potential (equation 1), which represents the interaction energy ($E_{DC}$) between the two systems as a function of the intermolecular distance $r_{DC}$:

$$E_{DC} = 4\varepsilon \cdot \left[ \left( \frac{\sigma}{r_{DC}} \right)^{12} - \left( \frac{\sigma}{r_{DC}} \right)^{6} \right] \quad (1)$$

Where ε and σ are the Lennard-Jones parameters to be identified through a best fit algorithm; they represent physical properties of the system: ε represents the equilibrium energy ($E_{DCmin} = -\varepsilon$), and σ gives the equilibrium length ($r_{DCmin} = \sigma \cdot 2^{1/6}$). The binding force ($F$) and the binding stiffness ($k$) are the first and the second order derivative of the interaction energy $E_{DC}$ with respect to the intermolecular distance $r_{CD}$ respectively, and they are expressed as:

$$F(r_{DC}) = -\frac{dE_{DC}}{dr_{DC}} = -24\varepsilon \cdot \left[ 2 \cdot \frac{\sigma^{12}}{r_{DC}^{13}} - \frac{\sigma^{6}}{r_{DC}^{7}} \right] \quad (2)$$

$$k = -\frac{d^2 E_{DC}}{r_{DC}^2}\bigg|_{r_{DC\,\min}} = \frac{72 \cdot \varepsilon}{r_{DC\,\min}^2} \qquad (3)$$

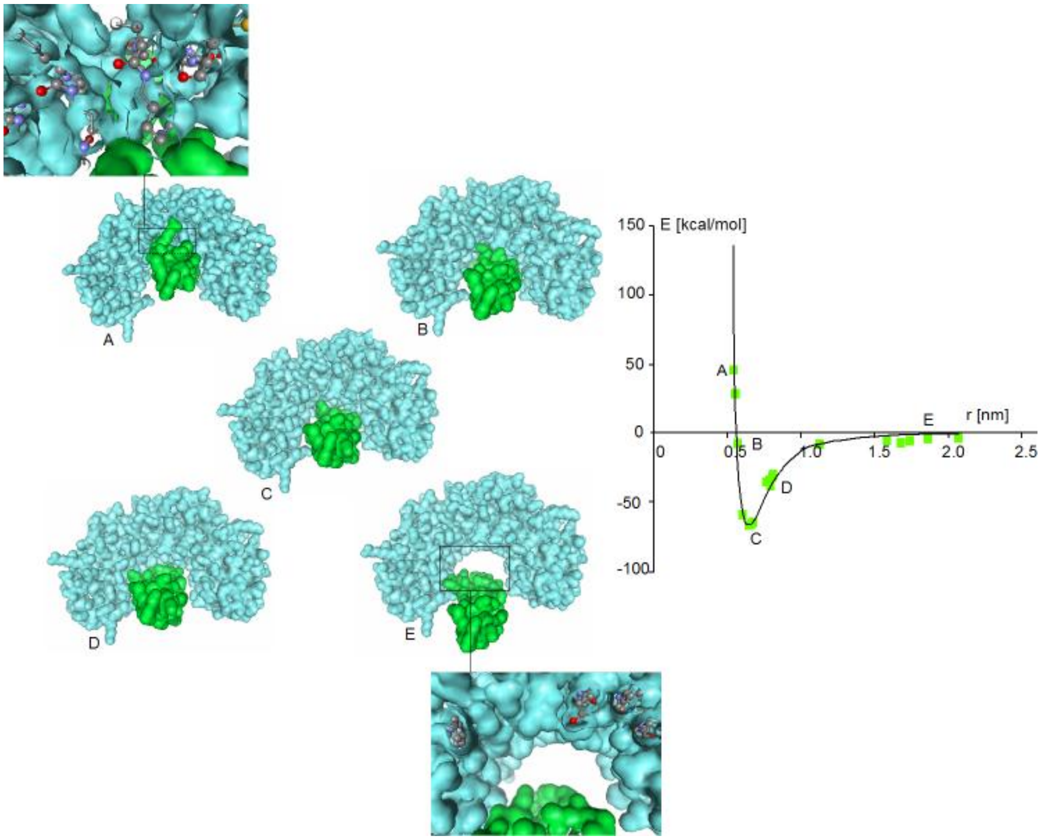

**FIGURE 2.** ***Collagen molecule-decorin core protein complex.*** The interaction between decorin core protein (in gray) and collagen molecule (in green) is evaluated for different intermolecular distances. The decorin concave surface is enlarged with reference to two simulations. Complex A is due to the collapse of the collagen within the decorin concave surface (highest energy value on graph), in the complex E decorin and collagen are too far and their interaction energy goes to zero (asymptotic value in the graph on the left).

The value for the binding stiffness calculated from the data obtained from the collagen binding site proposed by Keene et al. [8] was $8.62 \cdot 10^{-8}$ N/nm. The values calculated using the curve of the second binding collagen sequence were markedly lower, amounting to a stiffness of $1.54 \cdot 10^{-8}$ N/nm. These results indicate that the binding site proposed by Iozzo and co-workers[8] is more likely to be involved in decorin binding. The binding stiffness obtained for this binding site is three orders of magnitude greater then the earlier reported stiffness of the GAG chain[5]. Furthermore, the maximum bond strength of the decorin-collagen complex is larger then the ultimate strength of the GAG, implying that failure of the stress transfer system would proceed by bond cleavage in the GAG chain rather then by detachment of the decorin core protein.

The molecular modeling approach is a powerful tool to study the relationship between structure and function of a wide range of molecular complexes. For this approach some simplifications are required. In light of the recent publication by[14] the main approximation is likely to be the structure used for the decorin core protein taken from the RNAse inhibitor (in accordance to Iozzo's group), and not from Scott (dimeric form). In the case in which we adopted the second approach, some questions arose: Assuming that the dimeric structure is more stable in solution and that decorin PGs fasten along the fibril every 68 nm, *what is the molecular arrangement for the dimer which allows this requirement? What is the binding force between within the decorin and what is the binding force between the dimeric decorin and the collagen molecule?*

Another simplification concerns the environment used to model the molecular system; actually all molecular systems are surrounded by water molecules. However, a significant computational cost is associated with the large number of solvent molecules required to model a bulk solution. Alternatively, the solvent effect can be simulated with a continuum approach by accounting for its dielectric constant. This simplification was adopted in our simulations. With regard to collagen molecule modeling, as previously stated, collagen type I can be found in two distinct triple helix forms, this study focuses on the homotrimer one. In fact, this form represents only 5% of the total, and future studies can be focused on the heterotrimer form. However, according to the observation made by Keen and co-workers[8] that they found that decorin binds to the $\alpha_1$(I) chain, but not to the $\alpha_2$(I) chain, the homotrimer form adopted seemed adequate.

Molecular modeling is a powerful methodology for analyzing the three dimensional structure of biological macromolecules. There are many ways in which molecular modeling methods have been used to address problems in structural biology. This discipline includes all methodologies used in computational chemistry, like computation of energy of a molecular system, energy minimization or molecular dynamics. Computational methods to predict and analyze the structural and energetic properties of macromolecules and their interactions play an increasingly important role in a wide range of subjects such as biology, medicine, science materials, etc. The wealth of available sequences and structures provides an enormous database for computational efforts to predict structures, simulate docking and folding processes, simulate molecular interactions, and understand them in quantitative energetic terms. Nowadays, with the attention focusing on nano-problems, this approach can be also used to relate macroscopic behaviors with nanostructural features As stated in the second paragraph, tissues such as tendons and ligaments can be used as an ideal arena to perform studies aimed at the comprehension of the structure-function relationship of the molecular structures which form the ECM and together are the source of the mechanical properties of these tissues. Knowledge of these relationships can be useful in understanding connective tissue performance as a result of the cooperation and mutual interaction between different biological structures at the nanoscale.
